# Emerging evidence for preoperative anaemia screening and blood health optimisation for paediatric surgical patients

**DOI:** 10.1016/j.bjao.2026.100535

**Published:** 2026-02-10

**Authors:** Heidi M. Meyer, Susan M. Goobie

**Affiliations:** 1 Department of Anaesthesia and Perioperative Medicine, University of Cape Town, Red Cross War Memorial Children’s Hospital, Cape Town, South Africa; 2 Department of Anesthesiology, Perioperative and Pain Medicine, Boston Children’s Hospital, Harvard Medical School, Boston, MA, USA

**Keywords:** anaemia screening, paediatric anaesthesia, patient blood management, perioperative optimisation, preoperative anaemia

## Abstract

Preoperative anaemia is common in children presenting for elective surgery, including those without comorbidity, particularly in low- and middle-income settings. This editorial discusses emerging evidence on the prevalence of paediatric preoperative anaemia and considers the implications for anaemia screening and paediatric patient blood management pathways.

Preoperative anaemia is a significant and modifiable perioperative risk factor, associated with increased mortality, morbidity, and transfusion rates.^1^^,^^2^ The World Health Organization’s^3^ call to widely implement patient blood management (PBM) highlights the need for effective pathways to detect and optimise anaemia, whilst recognising the unique challenges presented both within resource-constrained settings and in the paediatric population. Evidence has shown that preoperative anaemia in children is common, even in high-income settings, with a higher prevalence in low- and middle-income countries.^1^^,^^2^ However, most available data are biased, only describing preoperative haemoglobin (Hb) values in children who have undergone preoperative testing, leaving the true prevalence among otherwise healthy elective surgical patients uncertain.

In this issue of *BJA Open*, Du Plessis and colleagues add evidence to the very limited data available from low- and middle-income countries on anaemia in children presenting for elective surgery within the public sector. The authors present a prospective cross-sectional study describing the prevalence of preoperative anaemia in ASA physical status classification 1 and 2 children aged between 6 months and 11 yr having elective surgery or diagnostic procedures at two tertiary hospitals in Johannesburg, South Africa. Point-of-care HemoCue® Hb analyser (HemoCue AB, Ängelholm, Sweden) testing was performed on venous blood samples in a cohort of 290 children with a mean age of 4.2 yr. The authors report an overall anaemia prevalence of 31.4%, with most cases classified as mild (57%) and only 2.2% severe. Screened children under 5 yr had the highest prevalence, consistent with global patterns.^4^ No associations were observed with nutritional status, sex, or recent deworming, although the study was underpowered to detect these. The strength of this study lies in its practicality, simplicity, and generalisability.

Limitations of the Du Plessis and colleagues work include accuracy constraints of HemoCue® as a point-of-care Hb screening tool. The authors used venous rather than capillary blood samples to improve accuracy. However, for large-scale implementation, capillary sampling remains more practical. This creates a potential gap between the results achieved and the realities of routine perioperative screening. Another limitation was the lack of a more accurate laboratory-based repeat Hb measurement which would have strengthened diagnostic confidence, particularly for values close to anaemia cut-off thresholds. However, this may have been impractical because of the additional costs involved and the extra blood draws in an already busy clinical environment. The authors’ pragmatic approach therefore remains consistent with real-world practice. The study by Du Plessis and colleagues also raises several unanswered questions. These include determining the true prevalence of anaemia using more accurate formal laboratory Hb measurement, understanding the underlying aetiology of anaemia in surgical populations, and establishing which children should undergo routine screening.

The high burden of anaemia observed in this paediatric cohort aligns with reports from Sub Saharan Africa, where up to 64.1% of children aged 6–59 months are anaemic.^5^ Although South Africa is classified as an upper-middle-income country, the public sector hospitals in which this study was conducted serve populations characterised by substantial socioeconomic disadvantage, marked inequality, and health system constraints that more closely resemble those seen in lower-income settings. These findings also echo the prevalence reported in the SAPSOS2 cohort (46.2%), which included children presenting for both elective and emergency surgery.^6^ The emerging pattern suggests that anaemia is not confined to specific high-risk surgical subgroups but is a recurring feature among children presenting for surgical care in environments where nutritional deficits, infectious diseases, and socioeconomic disparities are prevalent.

These results also prompt us to revisit an old question: should all children be routinely screened for anaemia before surgery, and if so, at what prevalence does universal screening become justified? Adult practice has shifted considerably over the past decade, with recognition of the prevalence and consequences of preoperative anaemia driving near universal screening, except in healthy young adults having low-risk procedures.^7^ In paediatrics, the issue is more complex, influenced by uncertainty regarding the optimal test, the limitations of point of care measurement, and the understandable reluctance to expose healthy children having surgery to an invasive blood test. These concerns must be balanced against the value of early detection, which informs perioperative risk assessment and creates opportunities for optimisation that extend beyond the operating theatre.

In contexts where anaemia prevalence is high, universal point of care screening may merit consideration as an alternative to risk-based approaches, particularly given the broader implications of anaemia for growth, development, and immunity. As with other screening strategies, the balance between benefit, cost, and unintended consequences is likely to be context dependent. In the South African public health sector, where access to primary healthcare is frequently constrained by high patient volumes, long waiting times, and challenges in continuity between community clinics and hospital services, the surgical visit may represent an important structured opportunity to identify and address reversible health risks before surgery.

This study highlights the potential opportunity to use the preoperative encounter as a point of contact for broader public health interventions, including deworming, iron supplementation, and nutrition counselling. These strategies are relatively inexpensive and scalable and align well with integrated approaches to child health. Cost, however, remains an important consideration. Adult PBM programmes, which incorporate optimisation of red cell mass, have demonstrated substantial cost savings,^8^ but equivalent economic evidence in paediatric populations is lacking, representing an important gap.

Looking ahead, several priorities for future research emerge. Implementation studies are needed to determine how screening strategies influence perioperative outcomes, workflow efficiency, and health system costs, and to identify which models are most feasible across diverse resource settings. Integrating Hb assessment into paediatric PBM pathways also requires careful attention to timing. Identifying anaemia should not automatically delay surgery but should inform decisions related to urgency, anticipated blood loss, opportunities for preoperative optimisation, and the need for postoperative monitoring. Evidence from perioperative settings suggests that simple point-of-care testing can reliably identify clinically meaningful anaemia even where laboratory infrastructure is limited, but its optimal role within paediatric pathways still requires clarification.^9^ Further work is needed to evaluate the cost effectiveness of universal compared with targeted strategies and to assess whether feasible and scalable interventions to optimise red cell mass translate into improved perioperative outcomes.

The study by Du Plessis and colleagues offers timely evidence that preoperative anaemia is common even among healthy children having elective surgery within the public sector in South Africa, reinforcing the growing appreciation that unrecognised paediatric preoperative anaemia is a significant concern worldwide. Although further work is required to define optimal screening strategies and to clarify the clinical implications of anaemia across diverse surgical contexts, the take home message is clear: incorporating simple, feasible Hb assessment into routine preoperative evaluation should be considered an important step toward improving perioperative optimisation and supporting child blood health more broadly. The emerging evidence positions preoperative anaemia assessment as a key element of paediatric perioperative care and underscores the need for context sensitive PBM pathways that are both feasible and equitable across different healthcare environments.

## Authors’ contributions

Conceived the editorial, interpreted the literature, drafted the manuscript, and led critical revision for intellectual content: HMM

Contributed to interpretation of the literature and critically revised the manuscript for important intellectual content: SMG

Approved the final manuscript and agree to be accountable for all aspects of the work: both authors

## Declarations of interest

SMG is Editor-in-Chief of *BJA Open*. HMM declares that they have no conflict of interest.
